# Correction to *Lancet Planet Health* 2023; 7: e219–32

**DOI:** 10.1016/S2542-5196(23)00083-9

**Published:** 2023-05-08

*Kliemann N, Rauber F, Bertazzi Levy R, et al. Food processing and cancer risk in Europe: results from the prospective EPIC cohort study.* Lancet Planet Health *2023; **7:** e219–32*—In table 3 of this Article, the association (hazard ratio) between percentage daily intake of NOVA 4 group food by mass and risk of oesophageal adenocarcinoma in model 2 should have been 1·20 (1·03–1·41). This correction has been made as of May 8, 2023.

